# CHARACTERISTICS AND INFLUENTIAL FACTORS OF ANXIETY AND BURNOUT AMONG CAREGIVERS AND OLDER ADULTS

**DOI:** 10.1093/geroni/igae098.2933

**Published:** 2024-12-31

**Authors:** Wenyu Zhang, Meihua Ji

**Affiliations:** Capital Medical University, Beijing, Beijing, China (People’s Republic); Capital Medical University, Beijing, Beijing, China (People’s Republic)

## Abstract

With rapid aging of the society worldwide, emerging research has suggested that mental disorders and high turnover among caregivers and older adults are substantial, however evidence in this area is limited. This study aimed to assess the characteristics and influential factors of burnout and anxiety among caregivers and older adults. A total of 184 caregivers, and 200 older adults from nursing homes as well as family settings were enrolled in this cross-sectional study. Participants’ job burnout and anxiety were assessed using the Maslach Burnout Inventory, and the Self-Rating Depression Scale, respectively, along with their sociodemographic information. Multiple linear regression was used to analyze the influential factors of job burnout in caregivers, and anxiety in both caregivers and older adults. As a result, there were 33.7%, 47.3% and 22.3% of participating caregivers reported high emotional exhaustion, depersonalization and low personal accomplishment, respectively. In terms of anxiety, 61.4% of caregivers reported their anxiety as moderate, 95% and 57% of older adults from nursing homes and family settings, respectively, were with moderate anxiety. Among caregivers, age, working hours, occupational stress and professional training significantly affected their level of burnout, while education and working experience prominently affected the level of anxiety. Meanwhile, the satisfaction to caregivers and self-care ability markedly affected the anxiety of older adults. In conclusion, there were high prevalence of job burnout and anxiety existed in both caregivers and older adults, more effective strategies should be taken into account to minimize this issue in the future.

